# House Drains

**Published:** 1873-04

**Authors:** 


### HOUSE DRAINS.

There exists a great danger from the use of drains as now arranged, connecting as they do, our water-closets, bathing-tubs and the kitchen sink, with the street sewer. From the decay of vegetable and animal matter, which naturally finds its way into the common sewer, noxious gases are formed, which, if allowed to escape into our living rooms, are capable of vitiating the air to that extent as to render it not only unwholesome, but absolutely dangerous. That this danger can and does occur, is readily demonstratible when observing the usual "water-trap," (which is intended to shut off the gases and foul air), during the sudden influx of the great volume of water that usually escapes into the sewer during a rain storm; when, the great pressure brought to bear upon the air and gases in the sewer, forces it through the drain pipe communicating with the house, and from thence through the water in your "trap," through which it bubbles into the room. Then, the difference of temperature between the air in the sewer and that in the dwelling, causes a current through the drain pipe into the house, again introducing the foul air of the sewer. Should a large quantity of water be allowed to escape,—as from a bath-tub, into a trap down stairs, every trap up stairs is likely to be emptied of its water through the suction induced by the vacuum which occurs behind the volume of water so escaping. There hen exists no impediment to free circulation of noxious gases into the chambers where such traps are located. In the covered cess-pools into which drains are usually conducted, the danger from escaping gases is far greater, as they must find an outlet somewhere, and naturally follow the drain pipe, into the dwelling, through the water in the trap. For this reason, cess-pools should be located at a considerable distance from the dwelling, and then have a suitable opening through its top, communicating with the open air, by means of a large pipe. Drains emptying into a sewer, should have a perpendicular pipe erected by the side of the house, inserted into the drain, and extend to the top of the house. By such an arrangement the gases would find unobstructed passage to the open air, when seeking such an outlet under the circumstances above numerated, and the foul air that we are now accustomed to recognize as escaping from our water traps, would find an exit far above our heads where their harmful influences would never be felt.

